# Household resilience of women migrant worker sellers of Jamu Gendong

**DOI:** 10.12688/f1000research.142709.1

**Published:** 2024-01-08

**Authors:** Dian Diniyati, Ary Widiyanto, Sanudin Sanudin, Eva Fauziyah, Budiman Achmad, Endah Suhaendah, Aditya Hani, Muhtar Muhtar, Danarti Danarti, Tri Sulistyati Widyaningsih, Aji Winara, Sri Najiyati, Rukmini Nugroho Dewi

**Affiliations:** 1 Research Center for Social Welfare, Village, and Connectivity, National Research and Innovation Agency, Jakarta, Indonesia; 2 Research Center for Population, National Research and Innovation Agency, Jakarta, Indonesia; 3 Research Center for Macroeconomics and Finance, National Research and Innovation Agency, Jakarta, Indonesia; 4 Research Center for Plant Conservation, Botanic Gardens, and Forestry, National Research and Innovation Agency, Bogor, Indonesia; 5 Research Center for Ecology and Ethnobiology, National Research and Innovation Agency, Bogor, Indonesia; 6 Research and Development Agency of West Java Province (BP2D), Bandung, Indonesia

**Keywords:** Jamu, livelihood assets, resilience, women

## Abstract

**Background:**

The selling of Jamu Gendong (an Indonesian traditional herbal medicine), is closely associated with the informal work of women who migrate to different regions. In Sukoharjo Regency and Wonogiri Regency, Central Java Province, the pressing need to meet household necessities in their places of origin compels women to assume the role of breadwinners. Therefore, this research aimed to identify the livelihood capital and resilience of migrant women selling jamu gendong.

**Methods:**

The study was conducted in Ciamis Regency, West Java Province, Indonesia, in November 2022, using quantitative and qualitative methods. Primary data were collected through structured questionnaires and in-depth interviews. The sample comprised 51 women selected through snowball sampling and actively involved in selling Jamu Gendong, along with six key individuals from relevant agencies.

**Results:**

The results showed that the households of migrant women selling jamu gendong survived and adapted to the migration destinations to prevent poverty. This was attributed to the livelihood strategy of business diversification. In this context, migration was exclusively practiced by women without family members who rented accommodation together. This research identified different forms of capital these women possess, including their productive age and skills in preparing jamu gendong, physical capital encompassing road infrastructure, marketplaces, access to health and education facilities, and natural, social, and financial capital.

**Conclusions:**

The findings contribute to a comprehensive understanding of the resilience demonstrated by migrant women selling jamu gendong. However, further research should be conducted in areas beyond the city center to obtain a holistic view of their resilience.

## Introduction

Migration is the movement of individuals or groups across spatial boundaries, involving a change of residence ( Kok, 1999). It is a global phenomenon that has been present throughout human history ( Castelli, 2018). People relocate for various reasons, including necessity, personal desire, or coercion, often driven by poverty or the pursuit of better economic opportunities in different locations ( Schrover, 2022). Approximately 258 million individuals migrate worldwide alongside considerable migration within individual countries, including Indonesia ( Azzam, 2017). Individuals who migrate are commonly referred to as migrants, indicating movement from a customary place of residence, within their country or across international borders ( IOM, 2023). Among the various regions in Indonesia, West Java stands out for having the highest number of internal migrants and is established as a prominent migration destination ( Badan Pusat Statistik Provinsi Jawa Barat, 2021). Ciamis Regency within West Java has attracted migrants from different origins, including Sukoharjo Regency, Surakarta City, and Wonogiri Regency in Central Java Province. These three regions have significant migrant populations, predominantly engaged in informal sector occupations within the destination areas, such as selling jamu gendong. Jamu gendong is a traditional Indonesian drink, generally made from fresh organic ingredients, in liquid form which is contained in several bottles then arranged neatly in baskets, and sold by carrying them in cloth by women who go around residential areas, markets and bus station ( Latifa *et al*., 2020; Limyati & Juniar, 1998; Pemerintah Kota Surakarta, 2022; Wulandari & Azrianingsih, 2014).

Selling jamu gendong is a form of informal work commonly practiced by women. According to Badan Pusat Statistik (2022b), the proportion of female informal workers (66.36%) surpassed that of their male counterparts (53.68%) in 2021. This phenomenon can be attributed to various factors, such as the husband’s low income, limited capital, low educational attainment, aspirations to improve the family’s economic situation, family business continuation, and social and environmental factors ( Mustofa *et al*., 2022).

Jamu gendong sellers migrate to expand their market reach, particularly in densely populated regions of Indonesia, such as West Java Province ( Badan Pusat Statistik, 2022a). Migration in Indonesia is motivated by several factors, including the pursuit of better job prospects and higher income to provide sufficient welfare for migrants and families, often in response to the lack of employment opportunities in their hometown ( De Royer *et al*., 2014), a desire to earn cash income ( Mulyoutami *et al*., 2015), greater employment opportunities outside the village, and the presence of friends or family members who extend invitations or have already relocated ( Fauziyah *et al*., 2015). The migration decision is influenced by age, land ownership, and income ( Wijaya & Syairozi, 2020).

To effectively adapt to the circumstances at the migration destination, jamu gendong sellers employ a livelihood strategy known as resilience ( Saraswati & Dharmawan, 2014). Resilience is defined as the capacity of a socioecological system to absorb disturbances, undergo changes, and retain its core functions, structure, and identity ( Nabin & Sgro, 2013). ( Grotberg, 1999) identified several sources of resilience, namely external support (I Have), individual ability (I Am), and social and interpersonal skills (I Can). Resilience encompasses the dimensions of resistance and toughness. It does not entail avoiding or eliminating adversity but rather involves navigating through challenges effectively and emerging stronger.

Resilience must be integrated with empowering and sustaining community livelihoods. The strategy encompasses constructing a comprehensive livelihood system of various methods to enhance life circumstances. This system must be supported by various livelihood assets ( Dharmawan, 2007a), including human, physical, natural, social, and financial capital ( Ellis, 2000a).

The households of female Jamu Gendong workers must compete with others to meet subsistence needs ( Badan Pusat Statistik Kabupaten Ciamis, 2023). The percentage of informal workers in Ciamis Regency is greater (69.48%) than formal workers (30.52%). This research identifies the livelihood capital and resilience possessed by migrant women selling jamu gendong in conducting their livelihood activities. The findings provide valuable insights into the experiences of migrant women, as the existing literature predominantly focuses on male migrants ( Schrover & Moloney, 2013). Migrant women remain largely overlooked, despite the significant impact of female migration on various aspects of their public and private lives, including worker participation, occupational distribution, religious beliefs, household responsibilities, and autonomy and self-esteem ( Pedraza, 1991).

## Methods

### Ethical considerations

This research has applied *Ethical approval* for research involving respondents. Ethical approval (ethical clearance) measures the ethical acceptability of a series of research processes. We obtained research ethical clearance approval from the BRIN Ethics Commission before the research began as a reference for upholding the values of integrity, honesty, and fairness in conducting research. In ethical clearance, there is informed consent, which guarantees the confidentiality of all information provided by participants. The ethical approval is included in the Ethics Commission for the Social Humanities Decree of the National Research and Innovation Agency No. 454/KE.01/SK/10/2022 dated 12 October 2022.

We also carried out the Consolidated Criteria for Reporting Qualitative Studies (COREQ) checklist before conducting the research. Regarding respondents and interviewer, we ensure that 1) there was no relationship established before study commencement; 2) participants know about the researcher’s reasons for collecting data; and 3) There was no bias, assumptions, reasons, or interests in the research topic for the interviewer/facilitator. Consent to be interviewed was given by the respondent in writing, by the respondent signing the questionnaire form, before the interview was conducted.

### Study design

**Figure 1.**
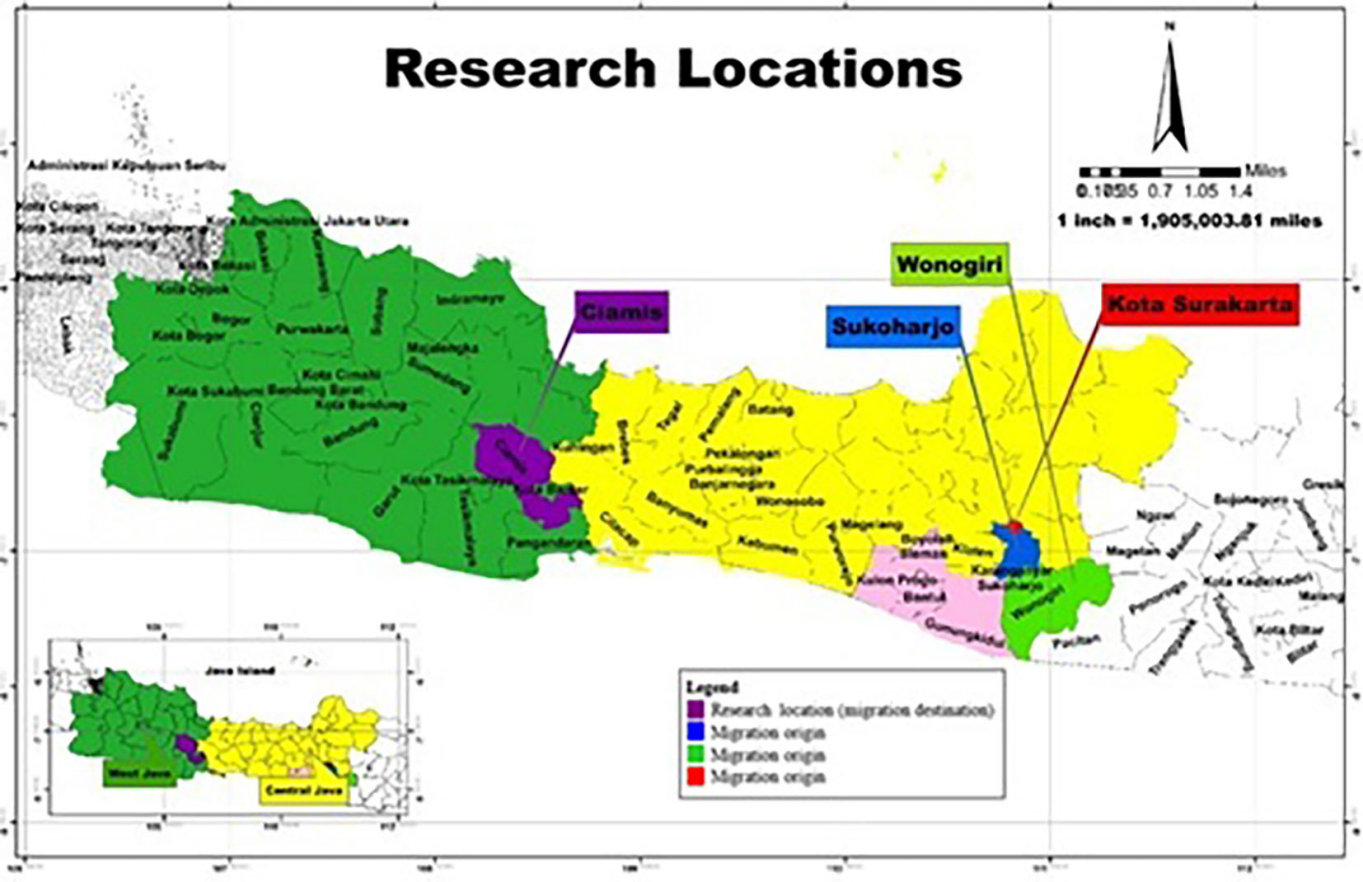
Research Location. (This figure is an original figure produced by the author(s) for this article).

This research used quantitative and qualitative methods to obtain more comprehensive, valid, reliable, and objective data ( Masrizal, 2012) in Ciamis Regency, West Java Province ( Figure 1). In November 2022, Ciamis Regency was selected as the research location because it is a destination for migrating residents outside the area who become informal workers selling jamu gendong, specifically from Sukoharjo Regency, Surakarta City, and Wonogiri Regency, Central Java Province.

The data collected comprised primary and secondary data. Primary data were collected through structured questionnaires and in-depth interviews using an interview guide. Structured interviews were conducted with respondents selected purposively by looking for jamu gendong sellers in shops. Secondary data was obtained from various sources such as documents and literature searches through websites related to the research. Literature is searched on several main indexers, namely Scopus and Google Scholar. Articles were searched using keywords such as “jamu gendong”, “herbal medicines”, “household resilience”, “migration”, “female worker”, and “livelihood”.

### Variables

Based on the research problems mentioned in the introduction, in this study research variables were compiled that influence the level of resilience of herbal medicine sellers as migrants. The level of resilience of households selling jamu gendong is influenced by the household income structure which includes a diversity of income sources. Ownership of assets by sellers of jamu gendong will influence the level of resale. The types of assets that will have an influence are Human Capital, Physical Capital, Natural Capital, Social Capital, Financial Capital.

### Questionnaire

This research used 51 participants who were selected by using snowball sampling. The participants were selected because of their experiences in selling jamu gendong for a minimum of two years. The research was conducted for the first time; therefore it used the specific questionnaires which were not used before. The suitability of questionnaires were evaluated before implemented in research activity. The final questionnaire and interview guide are provided as *Extended data* ( Widyaningsih, 2023).

### Interviews

In-depth interviews were conducted with six informants selected deliberately from related technical agencies, namely, officials at the Agriculture and Food Crops Agency of Ciamis Regency, Cooperatives, Small and Medium Enterprises, the Trade Agency of Ciamis Regency, the Regional Planning and Development Agency (Bappeda) of Ciamis Regency, West Java Regional VII Forest Service Branch with one of the working areas being Ciamis Regency, and the Center for Implementation of Environmental and Forestry Instrument Standards (BPSI LHK) Ciamis. Interviews were conducted with deliberately selected respondents. Determining respondents uses the snowball technique to identify the flow of data needed from one respondent to another.

Interviews were carried out by researchers who have more than 10 years of experience in social research. The total interview time was three months, depending on the respondent’s time availability. The majority of respondents were individuals, only a few were interviewed in groups. The interview media uses audio recordings, with prior approval from the respondent.

### Analysis

The Household Resiliency Index (HRI) is a simple, user-friendly tool that can be used to assess and classify households. The HRI has a quantitative questionnaire that assigns points to each response. The questionnaire includes variables that were carefully chosen to measure the impact of economic strengthening interventions on household assets, income, expenses, and health outcomes.

Three parameters are measured, i.e., asset and income, expenses, and health outcomes. Indicator for each parameter can be seen in Table 1, Table 2, and Table 3. Each parameter consists of several indicators which are scored according to the HRI guide for measuring household economic resilience developed by the Alliance for Child Protection in Humanitarian Action (2011). Assets and income = 35 points, including the indicator of monthly savings of max 10 points, livestock owned of max 10 points, and an increase in monthly income of max 5 points. Expenses = 35 points, including the number of meals per day max 10 points, ability to pay for basic needs max 10 points, ability to invest in income-generating activities (IGAs) max 15 points. Health outcome = 30 points, including Ability to pay for children’s school fees (level) max 10 points, Household members fully covered by health insurance max 5 points, Household food production level max 15 points. Decision criteria used as follows:

• Score 0-30 = Category 1 (household in destitution)
• Score 31-60 = Category 2 (Households Struggling to Make Ends Meet)
• Score 61-100 = Category 3 (Households Prepared to Grow)

**Table 1.** Indicators from assets and income.

Type	Indicator	Response	Points
Liquid asset ownership	Monthly savings (IDR/month)	a. No savings	0
		b. 100–9,999	5
		c. 10,000–19,999	10
		d. 20,000–50,000	15
		e. Above 50,000	20
	Owned cattle	a. Don’t have	0
		b. 1–9	5
		c. Ten or more	10
	Additional monthly income	a. Don’t have additional income	0
		b. Have additional income	5

**Table 2.** Indicators of expenses.

Type	Indicator	Index	Points
Food security	Number of meals per day	One time	0
		Two times	5
		Three times or more	10
Basic needs	Ability to obtain a basic income	Difficult to obtain	0
		Can be managed	5
		Easy to get	10
Investment	Ability to invest in others’ productive activity	Unable to invest	0
		Very difficult to invest	5
		Can be managed	10
		Easy to invest	15

**Table 3.** Indicators of household outcomes.

Type	Indicator	Index	Points
Education	Ability to pay school fees for children	Not attending school	0
		Primary school and middle school	5
		High school and above	10
Access to public health	Household members are fully covered by health insurance	No	0
		Yes	5
Food production	Ability to buy household food	Unable to buy food	0
		Able to buy food but not enough for household consumption	5
		Able to buy food and enough for household consumption	10
		Able to buy food in excess	15

After all the data was collected, the data was tabulated using the Microsoft Excel program for each parameter. The calculation results are then explained descriptively. Secondary data in related literature was used to strengthen, contrast, or compare research findings.

## Results

### A. Livelihood Structure of Female “Jamu Gendong” Sellers

**Figure 2.**
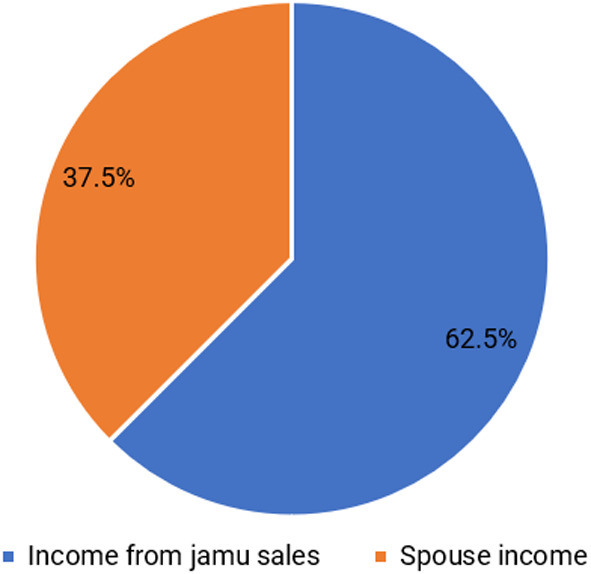
The proportion of main income and spouse income. (This figure is an original figure produced by the author(s) for this article).

Income structure is the composition of household income from various livelihood activities carried out by all household members ( Dharmawan, 2007b). Based on the results, the average income of respondents is IDR 55,664,492/year. The structure of the respondents’ income is divided into two groups: income from selling jamu gendong and income from spouses. Figure 2 shows that the family’s income is dominated by selling jamu gendong at 62.5%, while the remainder comes from the spouse’s income. Respondents use a dual income pattern in which husband and wife work together to earn a living to meet their needs ( Sumersi *et al*., 2021). According to Ellis (2000b), individuals and households diversify their livelihoods for two reasons, namely, need or choice.

**Figure 3.**
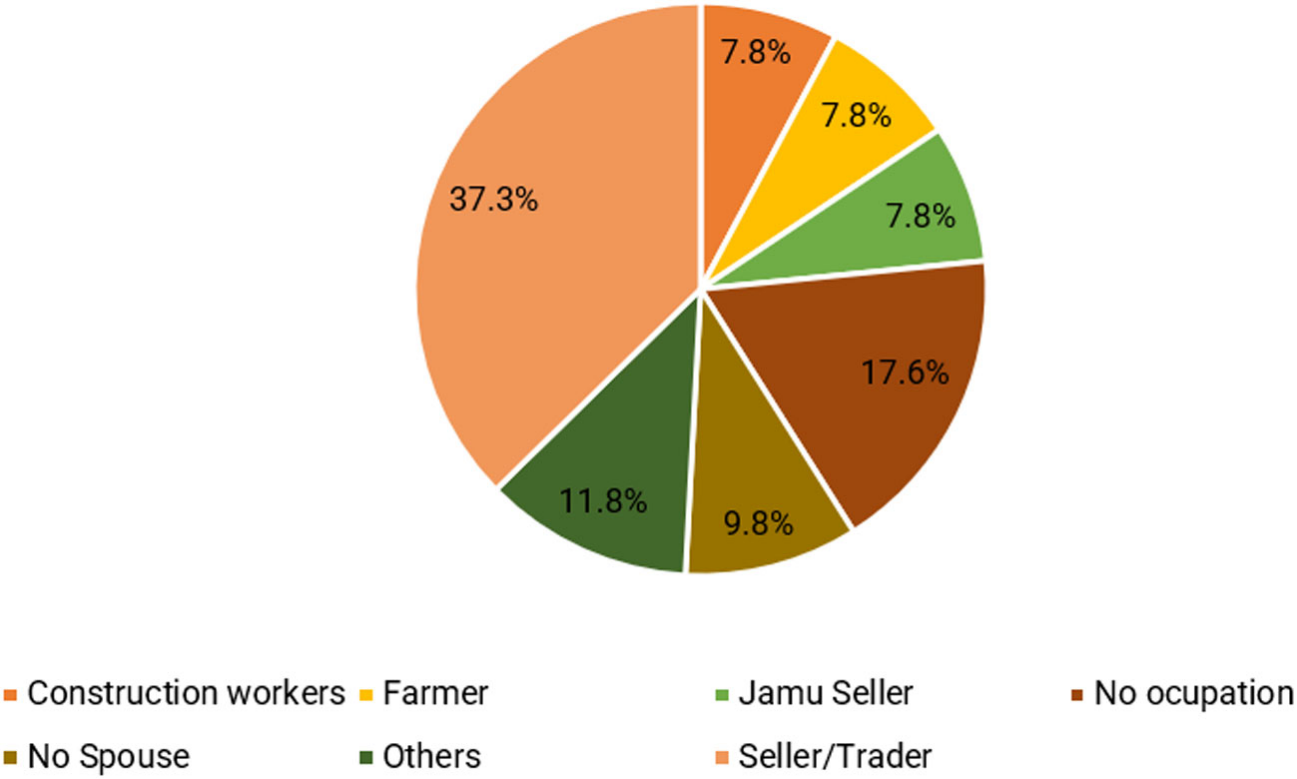
Type of work performed by the spouse. (This figure is an original figure produced by the author(s) for this article).

Furthermore, the couple’s primary occupation revolves around the role of sellers, with a particular emphasis on fast food, specifically meatballs. Five respondents (9.8%) did not have a partner, while nine (17.6%) had unemployed partners, resulting in a lack of supplementary income. The significance of women’s earnings as jamu sellers in bolstering family income is illustrated in Figure 3. By actively participating in the workforce, women effectively assist their husbands in sustaining the family’s economic stability and augmenting the total income ( Maradou *et al*., 2017; Musa *et al*., 2015), and providing a safety valve or household support to meet basic daily needs ( Ramli *et al*., 2020). Women’s involvement in economic activities must be acknowledged, even though there are differences in work between men and women.

According to Fridayanti and Dharmawan (2015) and Salatalohy (2019), income from a partner is part of a diversification strategy or a double income pattern ( Parker & Peterson, 2016; Smith, 2019). Even though the role of women is more dominant, the household income structure of jamu sellers can be categorized as a model of shared responsibility in which all members contribute to family income and share the responsibility for expenses. Each family member may have a source of income and contribute according to their means ( Johnson & Williams, 2018; Miller, 2017). The income is used to meet the family’s needs together ( Jackson & Davis, 2015; Miller, 2017). This model promotes participation and shared responsibility in managing family finances.

Household expenditure refers to all the expenses associated with purchasing goods and services to meet basic needs ( Illahi *et al*., 2018). These expenditures can be broadly categorized into food and nonfood expenditures ( Alfrida & Noor, 2017). Food expenditures typically include items such as rice, vegetables, side dishes, oil, etc. Nonfood expenditures include clothing, transportation, health, communication, water, electricity, taxes, LPG for cooking, and other nonfood consumption. 

According to the data presented in Table 4, the expenditure on food accounts for approximately 49.7% of household expenses, while nonfood slightly surpasses food expenditure at 50.3%. Trihastono *et al*. (2021) found that as household income increased, the proportion of spending on food tended to decrease, while the percentage allocated to nonfood items became larger. The higher the household income, the smaller the proportion of consumption allocated to food. Several factors influence the amount of household consumption, including economic, demographic, and noneconomic factors ( Illahi *et al*., 2018). Moreover, food expenditure serves as an indicator of economic well-being ( Haq, 2009). In societies where the percentage of spending on food is significantly lower than on nonfood items, greater prosperity is often observed. Household expenses for jamu gendong traders are shown in Table 4.

**Table 4.** Amount of Household Expenditures of Jamu Gendong Sellers.

No	Expenses	Amount (IDR/Year)	Percentage (%)
1	Consumption	15,077,551	49.7
2	Clothing	425,676	1.4
3	Electricity, water, taxes	1,764,337	5.8
4	Housing (rent)	2,697,875	8.9
5	The school (children)	4,091,000	13.5
6	Health	205,116	0.7
7	Remittance to family in hometown	3,191,149	10.5
8	Transportation	812,506	2.7
9	Communication	831,020	2.7
10	Social activity	607,778	2.0
11	Ventura capital	96,125	0.3
12	Others	527,027	1.7
	TOTAL	30,327,160	100.0

According to research conducted by the World Bank (2007), impoverished households in developing nations allocated a significant portion of their income, up to 70%–80%, toward food expenses. Expenditure on food purchased from jamu sellers accounted for nearly 50% of their income. Even though the World Bank (2007) considered this expenditure relatively modest, it remained the most significant component compared to others. The two primary nonfood expenses were directed toward educating children and providing transfers to families in their places of origin. Interestingly, this finding contradicted previous research that identified housing needs, such as rent or mortgage repayments ( UN-Habitat, 2003) and health costs ( World Health Organization, 2008) as the primary nonfood expenses.

The relatively smaller amount allocated to these categories can be attributed to the transfer activities directed toward families in their places of origin. Meanwhile, the monetary transfers made by migrant workers residing in cities are crucial in reducing poverty levels in their villages. The recipients of these transfers can utilize them to fulfill basic needs of food, housing, education, and health ( Rachmaniah & Suryahadi, 2016). They also underscore the benefits of migration, which provide village families with access to economic resources, information, and expanded social networks.

### B. Household Livelihood Assets of Women Jamu Gendong Sellers

Livelihood is defined as a means of earning a living ( Chamber and Conway, 1991). According to DFID (2001), sustainable livelihoods are needed for resilience, which is supported by five assets: human resources (capital), natural, physical, financial, and social capital. The livelihood assets owned by women selling jamu in Ciamis Regency are as follows.

1. Human Capital

Human capital encompasses the aptitudes, knowledge, capabilities, and good health that empower individuals to pursue diverse livelihood strategies and attain their objectives ( DFID, 2001). It refers to an individual’s intrinsic and acquired assets, including skills, abilities, and competencies ( Quandt, 2018). This concept entails proficiency, sound health, and the capacity to engage in various life strategies and endeavors to achieve livelihood goals ( Shen *et al*., 2009).

**Figure 4.**
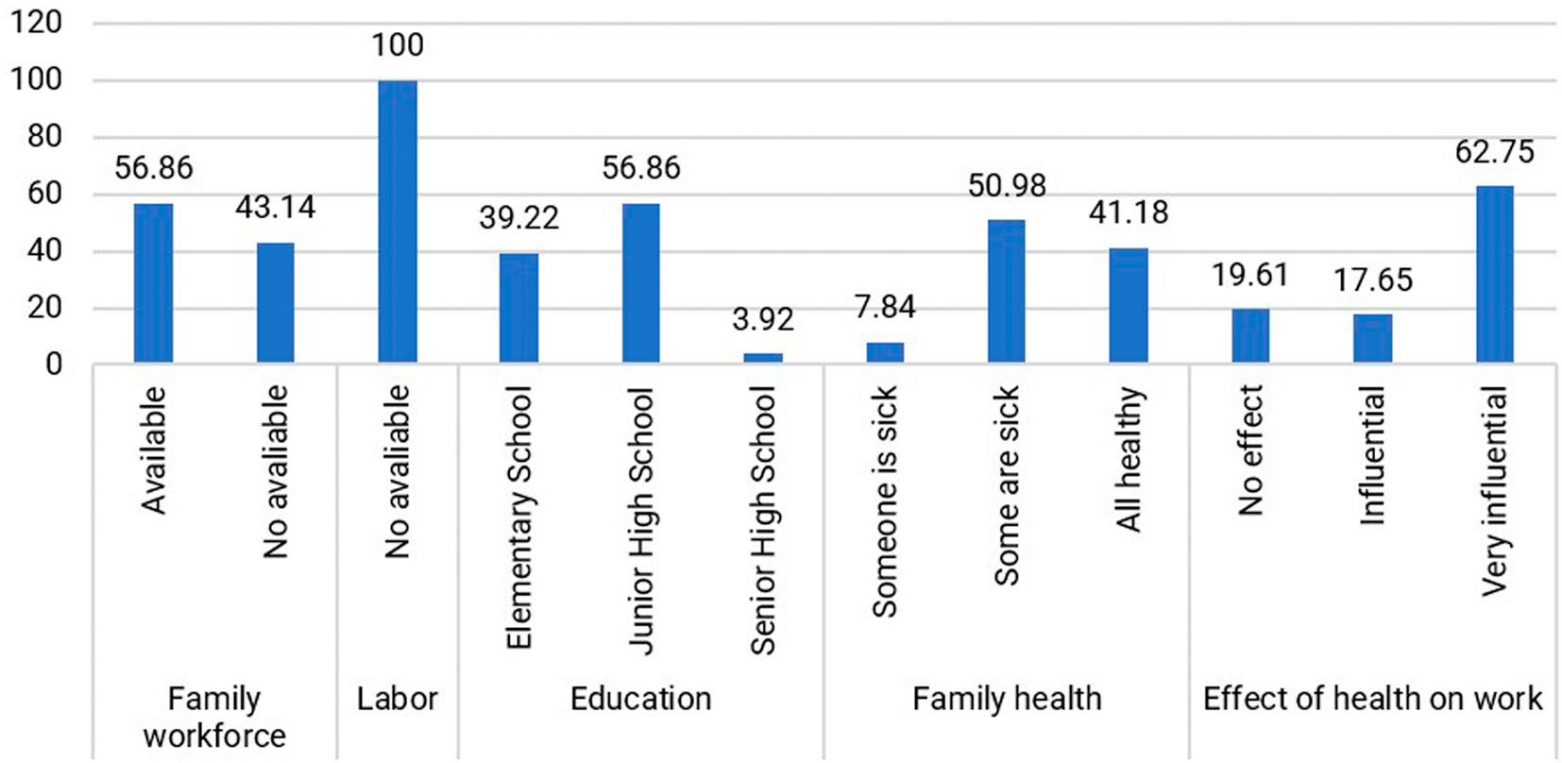
Distribution of respondents based on the condition of human capital. (This figure is an original figure produced by the author(s) for this article).

Within the jamu-selling community, individuals aged 40–49 years (47.06%) dominate the productive age group. Their education level predominantly comprises high school graduates (Junior High School and Senior High School), accounting for 60.78%, while those who did not attend or complete elementary school make up 39.22%. The level of education did not reduce the respondents’ interest in selling Jamu Gendong, as expressed by Mrs. Wn (54 years), who has been a seller since the age of 14: “I only went to school until I graduated from elementary school, but I have to sell jamu to help the family’s economy.” Therefore, the respondents are enthusiastic and motivated to survive in the face of economic shocks. They also have knowledge and skills in preparing jamu as a legacy from their ancestors that is still maintained. The distribution of respondents based on human capital is presented in Figure 4.

Assistance to Jamu Gendong sellers is primarily from family members, with 56.86% of respondents indicating that they receive support from their relatives. The remaining sellers handle the business themselves because they do not reside with family members. The absence of hired workers is not solely motivated by cost reduction but also by personal preference and the satisfaction derived from mixing the herbs. Beyene *et al*. (2023) stated that male-headed households were more resilient at meeting food needs. However, looking at the human capital condition of jamu sellers, women are also tough and play a significant role in meeting family needs. They encounter higher risks compared to their husbands, particularly in marketing. In addition to the necessity to visit customers, women are also susceptible to physical violence, such as robbery and verbal abuse ( Perry *et al*., 2020). Employment within the informal sector often fails to provide favorable conditions for decent work because it lacks security and legal protections. These sellers may lack proper channels or mechanisms to voice their grievances or assert their rights. The jamu sellers encounter challenges while selling that are often not reported to their husbands ( Vijayalakshmi *et al*., 2022).

2. Physical Capital

Physical capital comprises basic infrastructure and production goods needed to support livelihoods ( DFID, 2001). Infrastructure consists of changes in the physical environment that helps people meet their basic needs and become more productive. Produced goods are tools and equipment that people use to function more productively. Physical capital refers to access to services and infrastructure ( Adato & Meinzen-Dick, 2002), including skills, knowledge, education, health, and access to the family workforce ( Tacoli, 1999). It contributes to the local environment, including housing, public places, industries, bridges, dams, harbors, and shelters, as well as includes vital facilities such as electricity, water, telephone, and gas.

**Figure 5.**
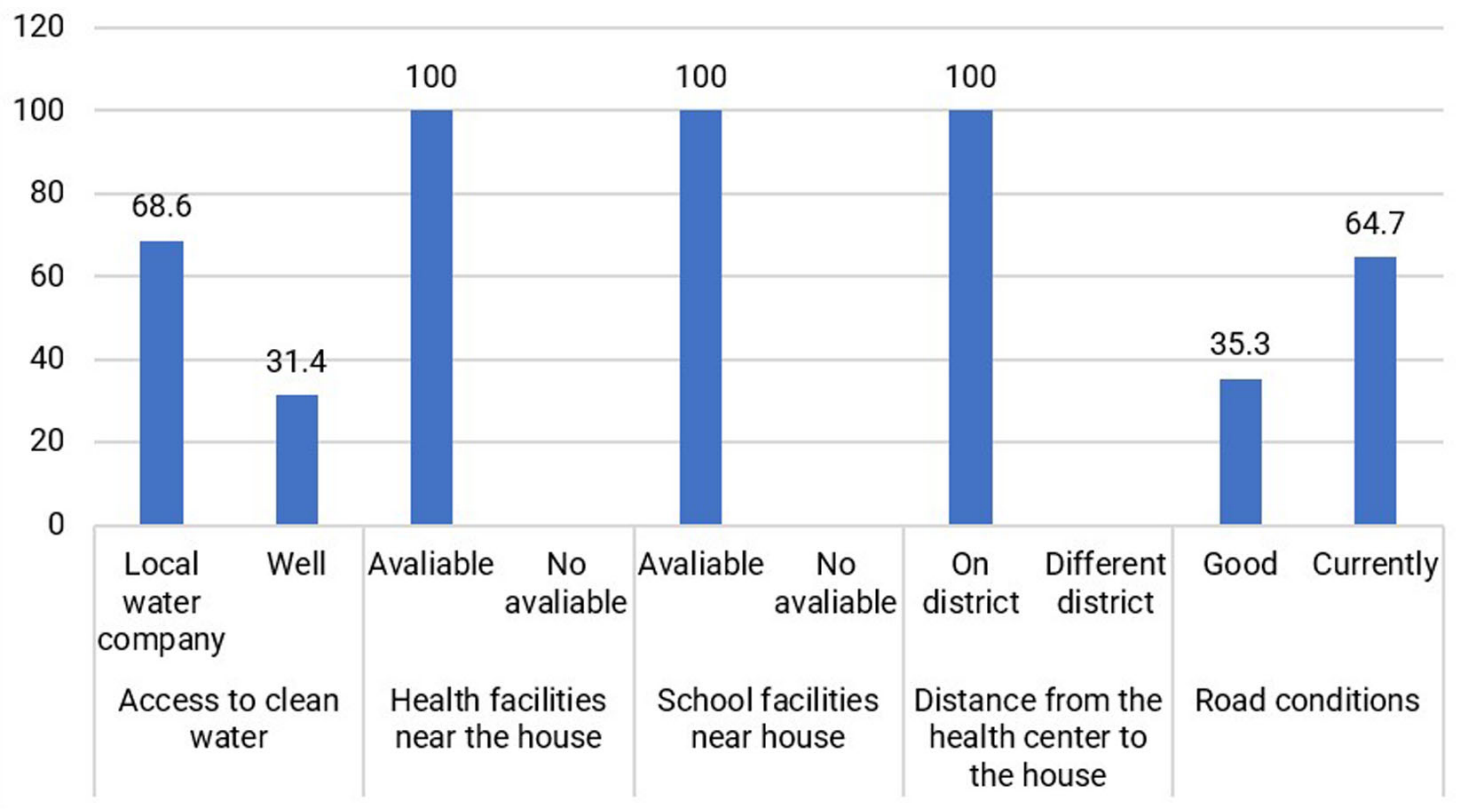
Distribution of respondents based on physical capital conditions. (This figure is an original figure produced by the author(s) for this article).

Based on Figure 5, the conditions of some of the Jamu Gendong sellers’ facilities are available at their place of residence, making it easier to live and conduct business. Generally, jamu sellers select to live in downtown Ciamis by renting small houses.

Jamu gendong vendors can capitalize on the existing physical infrastructure, including well-maintained roads and traditional markets that offer a wide range of equipment for producing and marketing jamu. Additionally, nearby health facilities are conveniently accessible, enabling jamu sellers to access necessary healthcare services. Educational institutions, such as schools, are also near their surroundings.

3. Natural Capital

**Figure 6.**
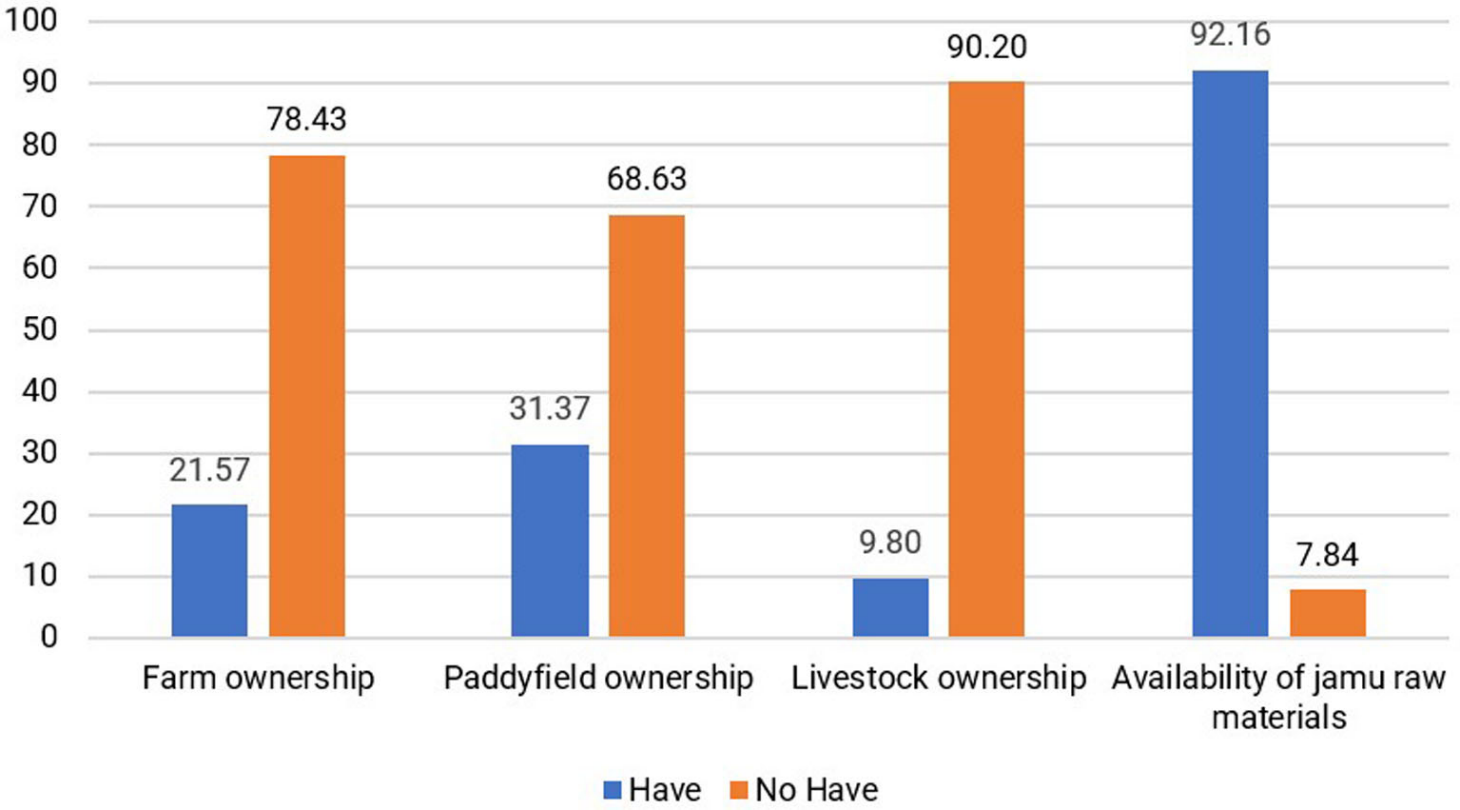
Distribution of respondents based on natural capital conditions. (This figure is an original figure produced by the author(s) for this article).

Natural capital refers to the inherent natural resources and elements that contribute to carrying capacity and provide valuable benefits to human livelihoods ( DFID, 2001). It holds particular significance for individuals who rely on natural resource-based activities as a primary or partial source of income ( Sharafi *et al*., 2018). These resources comprise various components, including land, water, mines, livestock, and other natural resources. Examples of natural capital are ownership of agricultural land, forests, pastures, water resources, and mineral industries ( Nasrnia & Ashktorab, 2021). Natural capital that generates carrying capacity and creates value for the livelihoods of jamu sellers includes the availability of raw materials for making jamu from the surrounding environment. According to 92.16% of respondents, there was no difficulty in obtaining raw materials. Raw materials can be obtained at traditional markets or from the gardens and rice fields of the surrounding community, as stated by 41.18% of respondents. Availability is the main natural capital for the resilience of the jamu business. Furthermore, other assets, such as gardens, rice fields, and livestock, are only owned by a small proportion of jamu sellers, as shown in Figure 6.

4. Social Capital

Social capital is a valuable resource used for pursuing livelihoods, encompassing networking, group affiliations, and mutual trust ( DFID, 2001). It comprises networks, groups, associations, relationships, trust, and interactions ( Adger *et al*., 2003). Individuals and communities can access resources and capital through communication and relationships. Social assets are the social resources people leverage to make a living ( Nasrnia & Ashktorab, 2021).

**Figure 7.**
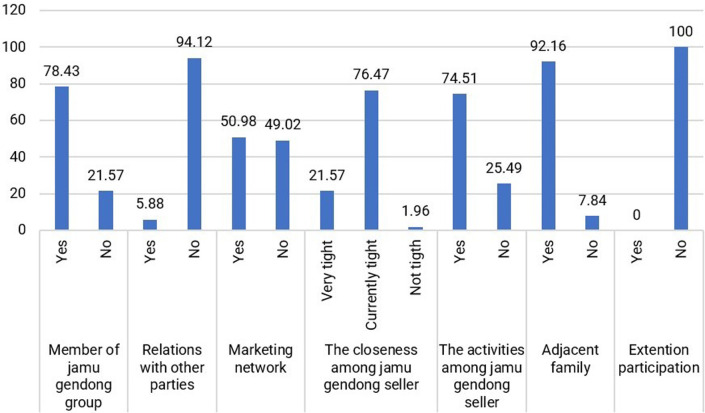
Distribution of respondents based on social capital. (This figure is an original figure produced by the author(s) for this article).

For jamu sellers, the social capital they develop primarily revolves around the community of fellow jamu sellers, which serves as a platform for interaction and problem-solving among peers. The physical proximity of their residences, indicated by 92.16% of respondents, enables close connections and fosters strong ties among jamu sellers ( Figure 7).

Financial capital refers to the resources that individuals use to achieve their life objectives. These resources encompass reserves or supplies that are owned by individuals or held by financial institutions. Financial capital includes a regular flow of funds, such as income, expenses, savings, accounts payable, and assistance ( DFID, 2001). It encompasses various forms of financial resources, including cash, bank accounts, savings, income, investments, credit, current assets, pension rights, allowances, grants, financial remittances, and household property ( Nasrnia & Ashktorab, 2021).

**Figure 8.**
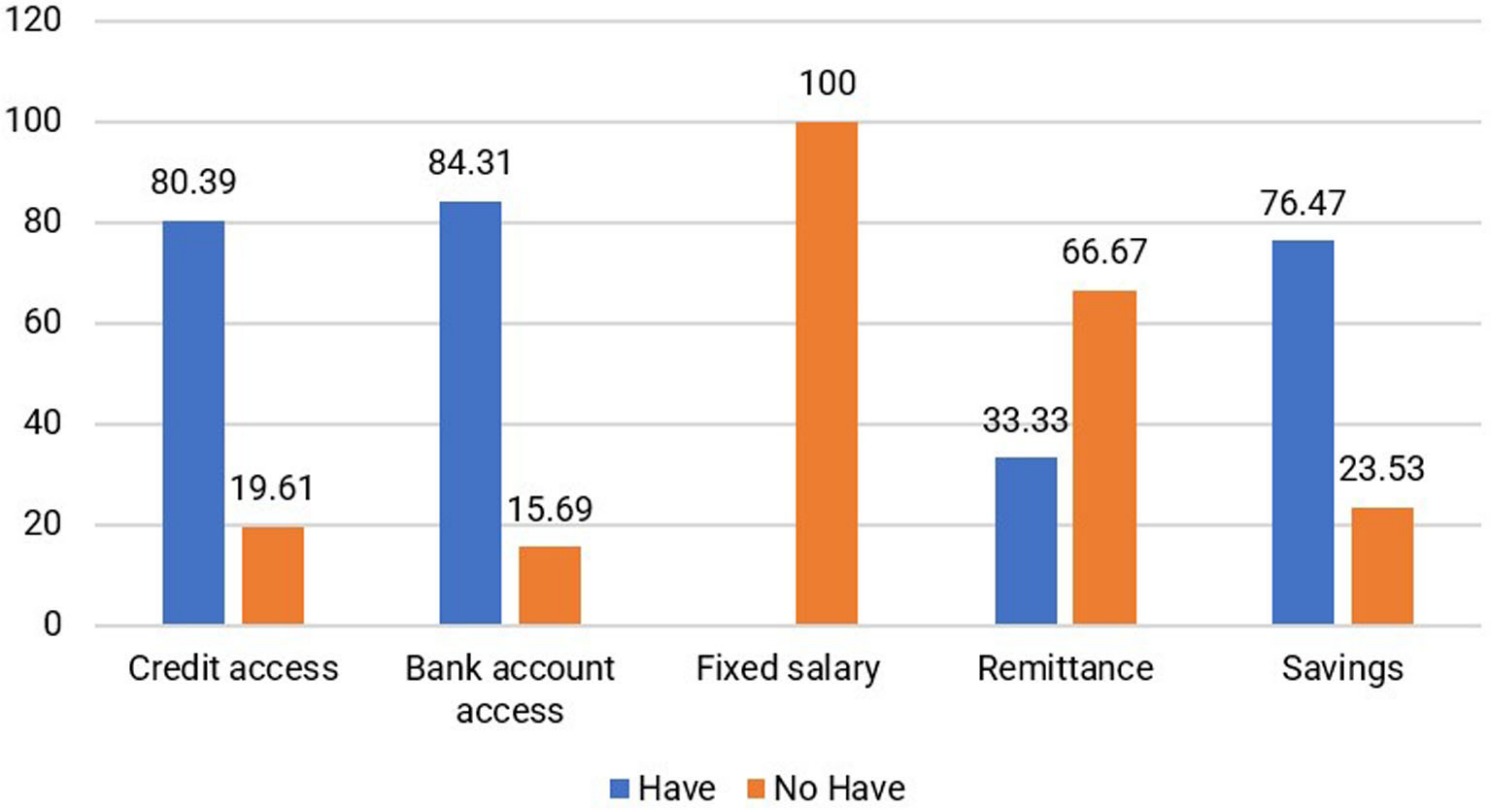
Distribution of respondents based on financial capital (This figure is an original figure produced by the author(s) for this article).

According to the findings, a significant majority of respondents assist their husbands while also engaging in additional income-generating activities, such as working as masseurs or selling meatballs and food. The financial assets are predominantly allocated toward household expenses, primarily to meet the needs of children and families residing in the area of origin. Furthermore, 76.47% of jamu sellers possess noncash savings, and 84.31% have bank accounts, as shown in Figure 8. Most respondents (80.39%) can access loans or credit from banks and other institutions. Households selling jamu have savings to use as needed. For instance, one of the respondents, Miss St (52 years old), mentioned that she not only saves in a bank account but also allocates a portion of her income toward building a house in her hometown and supporting her children’s education. Additionally, 21.57% own gardens and 31.37% possess rice fields in their hometowns. In addition to their role as natural capital, owning rice fields and gardens significantly contributes to the saving capacity of households.

## Discussion

Women selling jamu not only play a role in generating additional income but also build capital to survive and be able to face various crises. They can do so through access to a job (I Have), utilizing abilities (I Am), and the interpersonal skills developed by the jamu seller. The HRIs are ranked by 43 women jamu sellers (84.31%) in Category 2, eight (15.69%) in Category 3, and zero respondents in Category 1, as shown in Table 5.

**Table 5.** Average HRI value of Women Jamu Gendong Sellers.

Number of respondents	HRI C			HRI Total Value	Category	Remarks
	Assets and income	expenses	Health outcome			
0	0	0	0	0	1	The household is in squalor
43	10	15	20	45	2	Household struggles to make ends meet
8	20	30	20	70	3	Household is ready to grow

The resilience exhibited by the families of women who sell jamu is closely intertwined with the women’s lifestyles and work ethic. The inhabitants of Central Java are renowned for their strong work ethic and philosophical beliefs, such as “ *urip kudu dilakoni*” (life must be lived) and the necessity of being “ *gemi*” (thrifty). This philosophy reflects an optimistic outlook on life, enabling individuals to thrive in other communities and gradually achieve affluence ( Gianawati, 2017; Murdiyastomo, 2015).

The households of female jamu sellers in Category 2 struggle to make ends meet. These households have been able to meet their basic needs and have saved a limited amount. The savings are primarily in the form of cash, gold jewelry owned by the wife, motorbikes, and portfolio investments. However, these households encounter difficulties when it comes to investing in other productive ventures.

Among the respondents, eight households fall into Category 3, representing families poised for growth. These households have met their basic needs and also achieved some level of prosperity. The women are not solely focused on increasing their income but on cultivating skills, establishing connections, and possessing the necessary capital to invest in other businesses. They maintain physical assets and savings in their places of origin and engage in ventures in other communities, such as constructing rental properties and establishing savings and loan enterprises. Successful migrants typically expand their businesses promptly after accumulating capital ( Mulyoutami *et al*., 2015).

Household members in Categories 2 and 3 can easily access free health services through the Healthy Indonesia Card (KIS) program that aims to ease the burden on people with low incomes to access health services ( Simbolon *et al*., 2019). These households can also finance their children’s education beyond the educational background of their parents. According to Hazani *et al*. (2020), women’s contributions play a crucial role in family economies. On average, they effectively manage their time, balancing responsibilities within the household and in public settings. Women contribute to increasing the family’s income by assisting in meeting daily expenses, generating additional income, accumulating business and investment capital, saving, and covering costs related to healthcare and education.

Environmental changes ( Mallick *et al*., 2023) and limited resources affecting individuals, households, or social groups ( Mallick *et al*., 2023), along with the predominance of dryland agriculture resulting in insufficient agricultural production to meet household needs ( Nienkerke *et al*., 2023), have resulted in a decline in income. Consequently, individuals seek alternative sources of income, with migration being one of them ( Sobczak-Szelc & Fekih, 2020). Generally, migration occurs from regions experiencing low economic growth, with individuals seeking better livelihood opportunities to increase their family’s income. The household economic strategy for rural communities migrating to urban areas involves engaging in diverse economic activities among family members to establish multiple alternative sources of income and enhance resilience to change ( Hamidah & Santoso, 2021). In such cases, off-farm migration involves pursuing nonagricultural/nonland-based businesses.

Jamu sellers, who are predominantly immigrants, employ various strategies to augment their income and optimize household expenditures. This pattern is also evident among Subak farmers in Bali who migrate to cities, employing survival strategies through multiple income streams and developing social networks to bolster income and preserve their cultural heritage ( Darmawan *et al*., 2021). According to Hidayah *et al*. (2020), livelihood strategies carried out by households entail both economic and social strategies. The economic strategy is conducted by carrying out a double income pattern, using household workers and migration, while the social strategy is based on existing kinship ties.

Some of the strategies for fulfilling a living carried out by women selling jamu include:

1) Diversifying business activities, particularly by establishing dry food businesses, is important for households seeking to diversify their sources of income in both urban and rural areas ( Khan & Morrisey, 2023). Despite being engaged in jamu sales, which involve a manufacturing process lasting 1–3 h between 03.30 and 05.00 WIB, with most sales occurring between 06.30 and 11.30 WIB, jamu sellers still have available time to pursue additional income-generating work, such as selling dry food.
2) Migrating from one’s place of origin to another city without family members (husband and children). Family members remain in their place of origin to manage the land and assets, while the women who sell jamu return to their place of origin every month or two. This strategy is implemented to maintain normalcy and ensure that children can continue attending school in their home locality.
3) Living with a jamu seller who is a relative or neighbor from the same place of origin is a deliberate strategy aimed at facilitating coordination and communication. Additionally, it reduces expenses and increases savings that can be sent back to their places of origin as remittances. The presence of friends or relatives who have previously migrated to the same destination area plays a crucial role in establishing a network of migrants ( Massey *et al*., 1993).

In conclusion, the households of migrant women engaged in the sale of jamu were proven to have the ability to survive, improve their family’s economic conditions, and escape from poverty. Most of the Jamu Gendong sellers had left the impoverished conditions of households struggling to make ends meet, and a small number were ready to grow. This condition was supported by the ability to implement a livelihood strategy through business diversification. Migration was only carried out by women selling jamu, who rented a place to live together and combined it with a dual income strategy. The findings indicated that hard-working women overcame high economic and social pressures by developing strategies for economic survival and dealing with different challenges.

The living capital of migrant women selling jamu consisted of human capital in the form of productive age and skills in preparing jamu, physical capital in the form of roads, markets, health and education facilities, natural capital in the form of raw materials, social capital in the form of a community of Jamu Gendong sellers, and financial capital in the form of access to financial facilities. These five types of capital were owned and used by migrant women selling jamu in the form of a livelihood strategy.

It is recommended that the capabilities of these women be enhanced through training and counseling sessions focused on modern jamu processing techniques, disseminating accurate information regarding the proper utilization of jamu, and fostering marketing acumen. These recommendations should not be limited to the migration destination areas but should also extend to stakeholders in home areas.

## Data Availability

The interview transcripts cannot be shared publicly as they cannot be fully deidentified. Readers can request access from the corresponding author ( aryw002@brin.go.id). Figshare: Data Jamu Gendong.xlsx. https://doi.org/10.6084/m9.figshare.23798202 ( Widyaningsih, 2023). This project contains the following underlying data:
- Research questionnaire of Jamu Gendong.pdf - Interview guide of jamu gendong research.pdf Data are available under the terms of the Creative Commons Attribution 4.0 International license (CC-BY 4.0).
