# Supplementary material for: Household resilience of women migrant worker sellers of Jamu Gendong
Source: F1000Res. 2024 Nov 22;13:25. Originally published 2024 Jan 8. [Version 3] doi: 10.12688/f1000research.142709.3 (PMC11585856; doi:10.12688/f1000research.142709.3)
Supplement: Peer review history

# Reviewer report: Reviewer response for version 3

**DOI:** 10.5256/f1000research.174781.r342756

**Published:** 2024-11-23

**Authors:** Benni Hasbiyalloh

**Affiliations:** 1 Paramadina University, South Jakarta, Indonesia

The authors have addressed all my comments.

Is the work clearly and accurately presented and does it cite the current literature?

Partly

If applicable, is the statistical analysis and its interpretation appropriate?

I cannot comment. A qualified statistician is required.

Are all the source data underlying the results available to ensure full reproducibility?

No

Is the study design appropriate and is the work technically sound?

Yes

Are the conclusions drawn adequately supported by the results?

Partly

Are sufficient details of methods and analysis provided to allow replication by others?

No

Reviewer Expertise:

My area of research is Labour Migration and International Relations

I confirm that I have read this submission and believe that I have an appropriate level of expertise to confirm that it is of an acceptable scientific standard.

# Reviewer report: Reviewer response for version 2

**DOI:** 10.5256/f1000research.173463.r336148

**Published:** 2024-11-11

**Authors:** Benni Hasbiyalloh

**Affiliations:** 1 Paramadina University, South Jakarta, Indonesia

The article has improved significantly.

 The conclusion could be strengthened by addressing whether all questions have been answered. My focus is on question number three, which has not yet been adequately addressed in the conclusion. 

 To make the conclusion more comprehensive, you could briefly elaborate on how these strategies reflect resilience and are shaped by social capital and cultural values specific to their communities. This would ensure each question is addressed in a concise, focused manner.

 Also, in research problems, the authors mentioned that "the current literature tends to focus on men’s migration.". There are no academic sources cited to support the claims. Please include relevant literature so that others can evaluate the work.

Is the work clearly and accurately presented and does it cite the current literature?

Partly

If applicable, is the statistical analysis and its interpretation appropriate?

I cannot comment. A qualified statistician is required.

Are all the source data underlying the results available to ensure full reproducibility?

No

Is the study design appropriate and is the work technically sound?

Yes

Are the conclusions drawn adequately supported by the results?

Partly

Are sufficient details of methods and analysis provided to allow replication by others?

No

Reviewer Expertise:

My area of research is Labour Migration and International Relations

I confirm that I have read this submission and believe that I have an appropriate level of expertise to confirm that it is of an acceptable scientific standard, however I have significant reservations, as outlined above.

# Reviewer report: Reviewer response for version 2

**DOI:** 10.5256/f1000research.173463.r336147

**Published:** 2024-11-05

**Authors:** Md Imran Khan

**Affiliations:** 1 Jamia Millia Islamia, New Delhi, Delhi, India

It seems improved now. The author should write the conclusion as a separate section from the discussion.

Is the work clearly and accurately presented and does it cite the current literature?

Yes

If applicable, is the statistical analysis and its interpretation appropriate?

Yes

Are all the source data underlying the results available to ensure full reproducibility?

Yes

Is the study design appropriate and is the work technically sound?

Partly

Are the conclusions drawn adequately supported by the results?

Yes

Are sufficient details of methods and analysis provided to allow replication by others?

Yes

Reviewer Expertise:

I have observed overall aspect of the study. seem to be good. Need some changes

I confirm that I have read this submission and believe that I have an appropriate level of expertise to confirm that it is of an acceptable scientific standard.

# Response

**Published:** 2024-11-13

**Authors:** Ary Widiyanto

**Affiliations:** National Research and Innovation Agency Republic of Indonesia, Central Jakarta, Jakarta, Indonesia

Thank you very much for your review. We have separated the conclusion section. For the details, please check the revised manuscript.

# Reviewer report: Reviewer response for version 1

**DOI:** 10.5256/f1000research.156287.r326877

**Published:** 2024-10-03

**Authors:** Benni Hasbiyalloh

**Affiliations:** 1 Paramadina University, South Jakarta, Indonesia

While the study tackles an important and underexplored area of migration and gendered resilience, there are key aspects where the article could be improved for clarity, depth, and overall rigor. 

1. Conceptual and Theoretical Gaps
  - Differentiation of Migration Types: While the article introduces migration as a concept, it lacks a nuanced discussion of different types, such as circular, temporary, and permanent migration, which are crucial for understanding the mobility patterns and livelihood strategies of the jamu gendong sellers. Including these distinctions would provide greater conceptual clarity and strengthen the study’s theoretical grounding.
  - Coherence Between Theories and Analysis: The article mentions multiple theories and concepts (e.g., resilience, livelihood strategies, gender dynamics), but their connections to each other and to the findings are not always rigorously made. A clear conceptual framework outlining how these theories interlink and how they guide the analysis would enhance the study’s theoretical depth 
2. Integration of Gendered Dynamics
  - While the study focuses on women’s experiences in migration, the gendered aspects of resilience and economic roles are not sufficiently explored. A more thorough integration of a gender lens—examining how gender influences resilience strategies, social capital formation, and migration decisions—would provide a deeper understanding of the specific challenges and adaptations of female migrant workers.
3. Methods and Reproducibility Issues
  - Details on Sampling and Analysis Procedures: The article briefly describes using snowball sampling for both questionnaires and in-depth interviews, but further details on the selection criteria, diversity of respondents, and the flow of data collection would improve reproducibility. For instance, clarifying how initial participants were identified and ensuring diverse representation would strengthen the methodological rigor.
  - **Quantitative Sampling:** The use of snowball sampling for the 51 participants is noted, but further details would improve replicability. For instance:
    - How was the initial participant identified?
    - Were any inclusion/exclusion criteria besides having two years of experience applied?
    - Were any efforts made to ensure diverse representation (e.g., in terms of age, socioeconomic status)?
  - **Qualitative Sampling:** Six informants were selected from technical agencies, but the rationale for their selection is unclear. Provide more details on:
    - The specific criteria used to choose these informants.
    - How the snowball technique was applied in practice for the qualitative interviews.
  - While the HRI is explained, the exact process for scoring responses and interpreting results could be made clearer. Suggestions include:
    - Providing examples of how indicators are scored and weighted.
    - Explaining how the categories (0-30, 31-60, etc.) were developed and any theoretical or empirical basis for these thresholds.
4. Data Presentation and Analysis
  - The results are predominantly descriptive and do not always effectively link back to the theoretical framework. To provide a more robust analysis, findings should be clearly tied to the concepts of resilience, livelihood capital, and migration strategies, with discussions highlighting how empirical results support or contrast with existing literature.
  - The HRI is a potentially powerful tool for assessing household resilience, but its application is not clearly explained. For example, a step-by-step outline of how HRI scores were calculated and how households were categorized into resilience categories would greatly enhance understanding and enable other researchers to replicate the methodology.
5. Strengthening the Conclusions and Recommendations
  - The conclusions generally align with the findings but could be strengthened by explicitly stating how they address the research objectives outlined in the introduction. Additionally, the recommendations for practice—such as training and capacity-building for jamu sellers—should be tied directly to the study’s findings to ensure they are contextually relevant and actionable.
  - Highlighting the study’s unique contributions to the field (e.g., by emphasizing the gendered dimensions of informal migration and resilience) would provide a more compelling case for the significance of the research.

Is the work clearly and accurately presented and does it cite the current literature?

Partly

If applicable, is the statistical analysis and its interpretation appropriate?

I cannot comment. A qualified statistician is required.

Are all the source data underlying the results available to ensure full reproducibility?

No

Is the study design appropriate and is the work technically sound?

Yes

Are the conclusions drawn adequately supported by the results?

Partly

Are sufficient details of methods and analysis provided to allow replication by others?

No

Reviewer Expertise:

Migration Studies

I confirm that I have read this submission and believe that I have an appropriate level of expertise to confirm that it is of an acceptable scientific standard, however I have significant reservations, as outlined above.

# Response

**Published:** 2024-10-14

**Authors:** Ary Widiyanto

**Affiliations:** National Research and Innovation Agency Republic of Indonesia, Central Jakarta, Jakarta, Indonesia

**Article introduces migration as a concept, it lacks a nuanced discussion of different types, such as circular, temporary, and permanent migration article introduces migration as a concept, it lacks a nuanced discussion of different types, such as circular, temporary, and permanent migration.**

 Respond: This has been added in the introduction section (page 2)

This is known as circular migration, repeated, where herbal medicine sellers will return to their place of origin after temporarily living at their destination (Skeldon, 2012) . Herbal medicine sellers in Ciamis frequently perform this type of migration. However, some individuals carry out a temporary migration, namely moving for a certain period. After reaching their goal, they will return to their place of origin. Furthermore, permanent migration means moving to settle permanently in the destination. The last two models occur if the Jamu Gendong seller marries a resident.

**Coherence between theory and analysis &**

**examining how gender influences resilience strategies, social capital formation, and migration decisions**

 Respond: This has been added in the discussion section (page 11)

 . This study found that migrant women can exit from the limitation of financial capital by employing human capital, such as the skill to make jamu, physical capital, such as access to facilities and infrastructures, and social capital that links to other jamu sellers.

The women who sell jamu gendong in categories 2 and 3 have shown a capacity to handle their livelihood with resilience. Resilience is a significant factor in shaping the livelihood strategies of women selling jamu gendong, often limited by gender dynamics (Tanner et al., 2022) . Despite facing social and economic difficulties, women selling jamu gendong have demonstrated the ability to adapt and innovate their livelihood strategies, discovering ways to support their families. Women who sell jamu gendong have shown the capacity to empower themselves and disrupt traditional gender norms by implementing livelihood strategies, leading to fairer gender dynamics and more resilient future generations. The gender norm that women are sufficient to be housewives while household economics are the husband's responsibility (Happel & Becker, 1983), does not apply. Besides, men may have greater access to resources for long-distance migration or employment opportunities outside their immediate communities, whereas women’s networks may focus more on local support and survival strategies (Rica, 2008) . This cyclical relationship demonstrates how resilient and livelihood strategies can gradually alter gender roles, resulting in sustainable development and gender equality.

# Reviewer report: Reviewer response for version 1

**DOI:** 10.5256/f1000research.156287.r309665

**Published:** 2024-08-23

**Authors:** Md Imran Khan

**Affiliations:** 1 Jamia Millia Islamia, New Delhi, Delhi, India

The paper deals with an important area of research but needs some improvement to maintain the quality of work in this journal.

 1. The author should indicate the research problem and research questions in the methodology section.

 2. How this study contributes to the existing literature. Explain in the discussion section and abstract.

 3. The author has used the snowball sampling technique to collect the primary data, Explain in the methodology section how the result of this study is unbiased.

Is the work clearly and accurately presented and does it cite the current literature?

Yes

If applicable, is the statistical analysis and its interpretation appropriate?

Yes

Are all the source data underlying the results available to ensure full reproducibility?

Yes

Is the study design appropriate and is the work technically sound?

Partly

Are the conclusions drawn adequately supported by the results?

Yes

Are sufficient details of methods and analysis provided to allow replication by others?

Yes

Reviewer Expertise:

I have observed overall aspect of the study. seem to be good. Need some changes

I confirm that I have read this submission and believe that I have an appropriate level of expertise to confirm that it is of an acceptable scientific standard, however I have significant reservations, as outlined above.

# Response

**Published:** 2024-10-14

**Authors:** Ary Widiyanto

**Affiliations:** National Research and Innovation Agency Republic of Indonesia, Central Jakarta, Jakarta, Indonesia

**The author should indicate the methodology section's research problem and questions.**

**Respond**: Research problems and research questions have been added to the manuscript (page 4)

 Research problems

 Despite the importance of women's migration and their role in the informal sector, such as selling herbal medicine, for the economic sustainability of families and communities, the current literature tends to focus on men's migration. As a result, essential aspects of women's migration, including livelihood strategies, socio-economic resilience, and the influence of local social and cultural capital on migrant women's economic resilience, have not received adequate attention. Furthermore, there is still a lack of research on how migrant women in the informal sector, such as herbal medicine sellers, develop resilience strategies and use different forms of capital to survive in urban areas.

 Research Questions

 1.What strategies are used by migrant women who sell herbal medicine in urban areas to defend their lives and those of their families?

 2.Which types of capital (human, physical, social, natural, and financial) are employed by migrant women who sell herbal medicine to help them maintain their economic resilience?

 3.How do social capital and local cultural values influence migrants' livelihood strategies and financial security in the informal sector?

**How this study contributes to the existing literature. Explain in the discussion section and abstract.**

**Respond:** The ways to contribute to the literature were added in discussions and in the abstract.

 Page 10 (Economic resilience is not solely based on material resources, but also on the cultural values that the community adheres to)

 Page 11 (This study found that migrant women can exit from the limitation of financial capital by employing human capital, such as the skill to make jamu, physical capital, such as access to facilities and infrastructures, and social capital that links to other jamu sellers)

 Page 11 (This study provides a new perspective about business diversification in the informal sector (jamu gendong) and also adds insight into the livelihood strategies used by herbal medicine sellers to survive in difficult situations).

**The author has used the snowball sampling technique to collect the primary data, Explain in the methodology section how the result of this study is unbiased**

**Respond**: The steps to avoid bias of snowball sampling have been explained in the methodology section. More detail is showed in the manuscript (page 5)
